# Primary exophytic urothelial carcinoma of the bladder: A case report

**DOI:** 10.1097/MD.0000000000039187

**Published:** 2024-08-09

**Authors:** Liangliang Li, Kun Liu, Xueping Ma, Yameng Wu, Changxi Wang, Yangang Wang

**Affiliations:** a Department of Urology, The Fuyang Hospital of Anhui Medical University, Fuyang, China.

**Keywords:** bladder urothelial carcinoma, exophytic, no hematuria, partial cystectomy

## Abstract

**Rationale:**

Bladder urothelial carcinoma (UC) is a common urinary system tumor that is generally diagnosed by cystoscopy combined with pathological biopsy. However, complete exophytic UC of the bladder is very rare and difficult to diagnose. Early diagnosis and accurate identification of such tumors, followed by aggressive surgical treatment, is essential for the management of these patients.

**Patient concerns:**

An 84-year-old man was admitted to the hospital with dysuria, a poor diet, and significant weight loss.

**Diagnosis:**

Pelvic computed tomography and magnetic resonance imaging revealed an exteriophytic round mass on the right lateral wall of the bladder. Cystoscopy revealed a necrotic mass on the right lateral wall of the bladder cavity, and no tumor cells were found following the biopsy. The tumor was removed via partial cystectomy, and the pathological result indicated high-grade muscle-invasive UC.

**Interventions:**

The patient refused radical cystectomy and underwent laparoscopic partial cystectomy plus pelvic lymph node dissection followed by cisplatin plus gemcitabine chemotherapy.

**Outcomes:**

The patient’s mental state and appetite were significantly improved after the urinary tube was removed 1 week after surgery. His general state was significantly improved after 1 month of follow-up but died of acute cerebral infarction 3 months after surgery.

**Lessons:**

UC of the bladder may grow completely out of the bladder without symptoms such as gross hematuria; thus, early diagnosis is difficult. For high-risk individuals, regular imaging tests may help to detect tumors early. Partial cystectomy is a reliable surgical modality for bladder preservation in such patients.

## 1. Introduction

Bladder cancer is the second most common urological malignancy worldwide, and its histological types of bladder cancer mainly include urothelial carcinoma (UC), squamous cell carcinoma, and adenocarcinoma.^[1]^ Bladder UC originates in the bladder urothelium and most of these tumors grow into the lumen. Muscle-invasive UC can invade the detrusor muscle and even grow out of the bladder, and its prognosis is relatively poor. However, complete exophytic UC of the bladder is rare in the clinical setting. In this study, an elderly man with no history of gross hematuria was admitted to the hospital because of dysuria. An exophytic mass was found on the right lateral wall of the bladder, which was eventually diagnosed as high-grade muscle-invasive UC after surgical resection.

## 2. Case presentation

**Figure 1.**
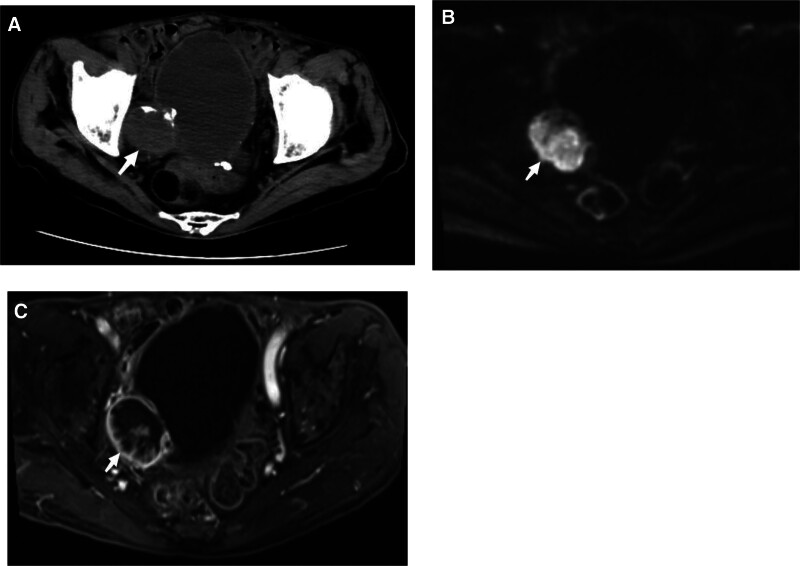
Radiographic features of the patient. (A) Pelvic CT: A mass (arrow) of approximately 34.9 × 25.7 × 35.2 mm was found on the right side of the bladder, and the mass was exophytic and partially calcified inside. (B) Pelvic MRI: the mass (arrow) had a high diffusion-weighted signal. (C) Pelvic MRI: the mass (arrow) was significantly enhanced on the dynamic enhanced image.

**Figure 2.**
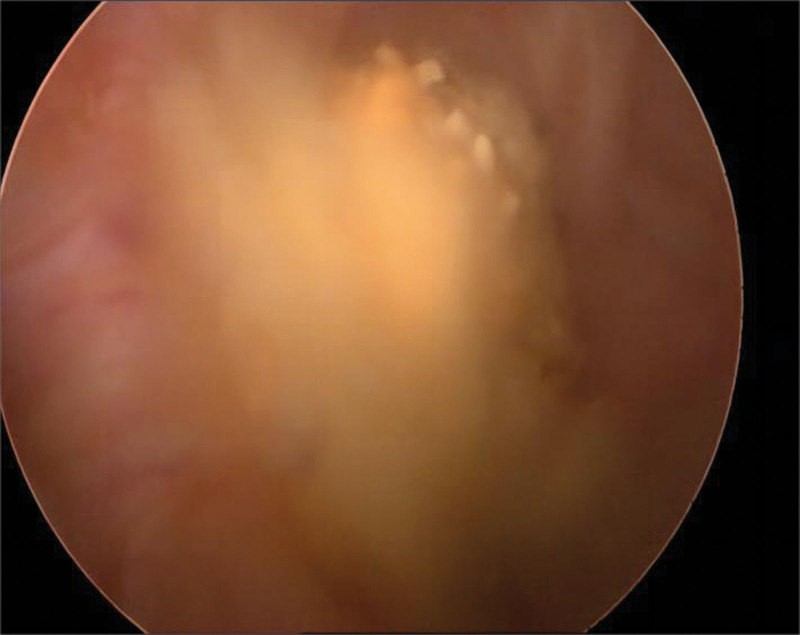
Cystoscopy revealed a 6 × 6 mm yellow necrotic mass on the right lateral wall of the bladder, and no obvious bleeding was found.

**Figure 3.**
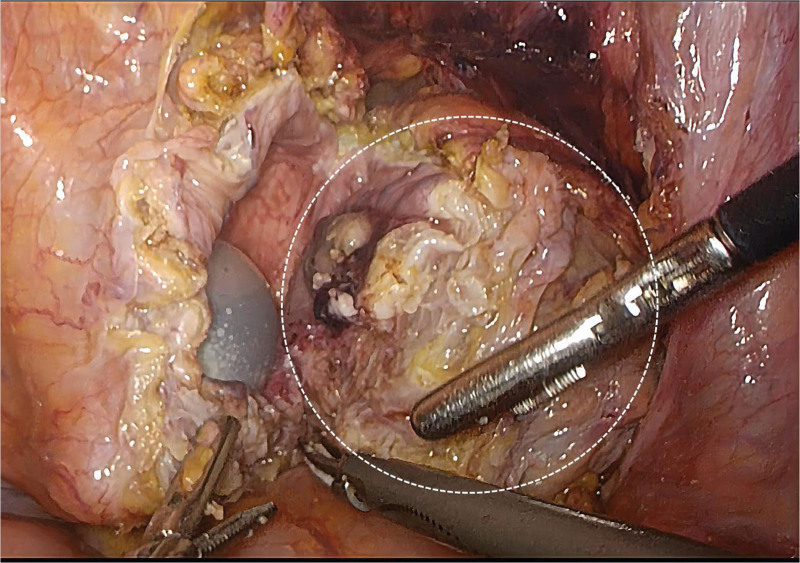
During partial cystectomy, it was found that the tumor (dashed circle) grew outside the bladder wall, was clearly outlined, and the extravesical peritoneum was intact without violation.

**Figure 4.**
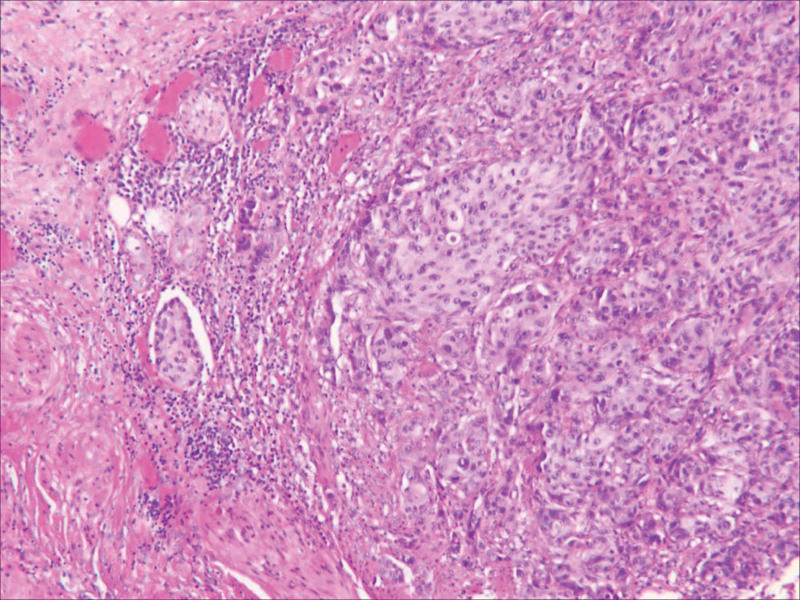
Histopathological examination revealed a high-grade muscle-invasive urothelial carcinoma (hematoxylin and eosin stain, original magnification, ×100).

An 84-year-old man presented to the hospital with urination difficulties for 1 year, and his symptoms had worsened over the previous month. This was accompanied by a poor diet and a weight loss of approximately 10 kg. The patient had a history of intracerebral hemorrhage and no history of smoking or alcohol consumption. On physical examination, the abdomen was soft and flat with no suspicious palpable mass or tenderness. Computed tomography (CT) revealed an exteriophytic round mass on the right lateral wall of the bladder 34.9 × 25.7 × 35.2 mm in size (Fig. 1A). The patient did not have a history of gross hematuria, and 21 red blood cells/μL (reference range: 0–15 cells/μL) were found on urine analysis. Pelvic magnetic resonance imaging (MRI) revealed that the mass had a high diffusion-weighted signal (Fig. 1B), which was significantly enhanced in the dynamic enhanced image (Fig. 1C). Cystoscopy revealed a yellow necrotic mass, approximately 6 × 6 mm in size, on the right lateral wall of the bladder (Fig. 2), and biopsy showed no definite tumor cells. The patient’s general condition was poor, with a body mass index of only 16.4 kg/m^2^. He refused radical cystectomy and underwent laparoscopic partial cystectomy. Intraoperatively, the tumor grew outside the bladder and was clearly demarcated from the surrounding tissue (Fig. 3). Postoperative pathology revealed a high-grade muscle-invasive UC (Fig. 4).

## 3. Discussion

Bladder cancer is one of the most common malignant tumors worldwide, ranking 10th in terms of incidence.^[2]^ More than 90% of the histological subtypes are UC, which can be divided into non–muscle-invasive bladder cancer and muscle-invasive bladder cancer (MIBC).^[3]^ About 25% to 30% of bladder cancers are first diagnosed as MIBC, which has a higher propensity to spread to lymph nodes and other organs and has a poor prognosis. The 5-year overall survival rate of patients with MIBC is approximately 60% to 70%.^[4,5]^

Bladder UC mostly grows in the cavity, and the most common symptoms are gross hematuria or microscopic hematuria, accounting for 78.3% and 13.7% of cases, respectively.^[6]^ Most exoplastic tumors are locally advanced tumors that mainly invade the surrounding tissues and organs, but bladder UC with complete extraluminal growth is rare in clinical practice. In the present case, a mass was found on the right wall of the bladder due to prostatic hyperplasia combined with dysuria. The mass was located outside the bladder wall, and a yellow necrotic mass on the right lateral wall of the bladder was found by cystoscopy, which was completely different from the common UC. No tumor cells were found on postoperative pathological examination of the biopsy specimen. This resulted in diagnostic difficulties, and we further examined the chest and abdominal CT scans to rule out the possibility of metastasis. Both pelvic CT and MRI indicated the possibility of bladder malignancy; therefore, radical cystectomy was initially considered. However, due to the patient’s poor physical condition with a body mass index of 16.4 kg/m^2^, a strong request for preservation of the bladder, and impossible transurethral resection of the bladder tumor (TURBT) as the tumor was located outside the bladder cavity, we performed laparoscopic partial cystectomy. During the operation, the tumor was found to be growing on the right wall of the bladder, had a clear boundary with the surrounding tissues, and did not invade the surrounding organs, which was not consistent with the usual presentation of invasive UC. Therefore, the tumor was not considered UC. The tumor and surrounding fat were completely removed, and pelvic lymph nodes were dissected. Postoperative pathology confirmed that the tumor was high-grade muscle-invasive UC, and lymph node metastasis was not found.

At present, preoperative neoadjuvant chemotherapy combined with radical cystectomy remains the preferred treatment for MIBC.^[7]^ However, for patients with MIBC who are physically unable to tolerate radical cystectomy or unwilling to undergo radical cystectomy, comprehensive treatment with bladder preservation based on TURBT can be considered.^[8]^ However, for some tumors in special sites, some studies have shown that partial cystectomy has reasonable cancer control rates and will not affect the prognosis of tumors.^[9,10]^ In this case, the tumor type could not be confirmed before surgery, and the patient refused bladder removal; therefore, bladder preservation surgery was performed. As the tumor was located almost entirely outside of the bladder cavity, TURBT was not feasible. Therefore, partial cystectomy was selected as the best surgical option for bladder preservation. The patient recovered well after the operation, and his diet and weight significantly improved compared with those before surgery. The patient was administered cisplatin combined with gemcitabine chemotherapy 1 month after the operation; however, he could not tolerate this regimen and developed related adverse reactions. Therefore, further treatment was discontinued. Unfortunately, the patient died of acute cerebral infarction approximately 3 months after surgery.

Primary bladder UC is a common malignant tumor of the urinary system and cystoscopy combined with pathological biopsy remains an essential diagnostic method.^[1,11]^ However, the growth pattern of the bladder tumor in this patient was different from that of common UC and was almost completely exogenous; thus, it was difficult to confirm the diagnosis before tumor resection. Primary exophytic tumors of the bladder are rare, and common exophytic tumors are generally tumors of relatively special tissue types, such as urethral adenocarcinoma, leiomyomas, and plasmacytoid UC. However, they differ from exophytic UC in terms of location and growth mode.^[12–14]^ Although the tumor in this patient was large and invasive, it did not invade any tissue or organ, and no tumor was found, even in the fat around the bladder. We believe that this was related to the existence of structures similar to false envelopes around the tumor; however, as no relevant studies have previously been reported, the specific cause is unknown. Further in-depth studies on such tumors are required in the future.

Complete external bladder UC is rare, and there are no obvious positive signs such as gross hematuria in the early stage. Diagnosis can be easily missed and delayed. Our patient is an elderly patient with bladder outlet obstruction, so exophytic tumors do not exclude urothelial cancer arising in the diverticulum of the urinary bladder, which is not clear during our diagnosis and treatment. In addition, a simple cold cup cystoscopic biopsy may not provide sufficient tissue for histopathology, and formal transurethral loop resection of the tumor will be better used to confirm our diagnosis. These deficiencies in our diagnosis and treatment process will provide a better experience for similar medical records in the future.

## 4. Conclusions

As there are no positive signs of complete exophytic bladder UC, it is difficult to diagnose it early, and the screening of high-risk groups needs to be strengthened, especially the application of CT and MRI, to facilitate early detection, diagnosis, and treatment. In terms of treatment, bladder preservation therapy with partial cystectomy as a surgical option is worthy of affirmation for tumor control. This report provides a new understanding of the specific growth pattern of bladder UC and evidence for future clinical diagnosis and treatment.

## Author contributions

**Conceptualization:** Liangliang Li, Yameng Wu.

**Writing—original draft:** Liangliang Li.

**Writing—review & editing:** Liangliang Li, Yangang Wang.

**Data curation:** Kun Liu.

**Formal analysis:** Xueping Ma.

**Resources:** Changxi Wang.
